# Experience With the Use of Intraoperative Diagnosis as an Aid to Definitive Diagnosis in Malignant Lesions: A Retrospective Study From a Tertiary Cancer Institute

**DOI:** 10.7759/cureus.109833

**Published:** 2026-05-28

**Authors:** Priyanka Sameer, Deepti Mishra, Priyadarshini Guha, Shreshtha Ghosh, Somdatt Sen

**Affiliations:** 1 Department of Pathology and Cancer Genetics, Kalyan Singh Super Speciality Cancer Institute, Lucknow, IND

**Keywords:** accuracy, diagnosis, intraoperative frozen section, margin status, sensitivity

## Abstract

Background

Intraoperative frozen section (IFS) helps the surgeon in surgical management by providing intraoperative diagnosis of tumors, assessment of margins and lymph nodes, and identification of metastasis. The present study aims to evaluate the indications for IFS consultation and determine its diagnostic accuracy across major anatomical sites to improve clinical management.

Methodology

This retrospective study included 228 IFS samples over two years (2023-2024). The histopathology findings of IFS were compared with corresponding formalin-fixed paraffin-embedded (FFPE) tissue, and the results were categorized into concordant and discordant. The causes of diagnostic differences were systematically analyzed. Diagnostic parameters, including accuracy, sensitivity, specificity, error rates, indication, tissue types, and limitations, were also evaluated.

Results

In the present study, the number of female patients (146, 64%) was higher than that of male patients (82, 36%), and the age range of patients was between 10 and 74 years. The maximum frozen section (FS) sample for diagnosis was obtained from the breast (59, 25.8%), followed by the ovary (45, 19.7%), head and neck (44, 19.3%), lymph nodes (31, 13.6%), omentum (13, 5.7%), gallbladder (12, 5.3%) and other anatomical sites (24, 10.5%). The most common indication for FS examination was margin assessment (107, 46.9%). In a review of nine discordance cases, misinterpretation error was found in five cases, sampling errors in three cases, and technical errors in one case. The overall diagnostic accuracy of FS was 96.1%, with a sensitivity of 90.2% and specificity of 99.3%.

Conclusions

The study demonstrated that FS is a useful technique for evaluating pathological entities and aiding in the surgical management of patients. It also helps identify areas requiring improvement to reduce error rates in FS.

## Introduction

Intraoperative frozen section (IFS) plays a crucial role during surgery by providing instant pathological information for the rapid diagnosis of suspicious tissue masses. It is most often performed in oncological surgery to determine the nature of a tissue sample, differentiate between benign and malignant samples, evaluate the surgical margin of the tumor, and detect metastasis in lymph nodes [1]. Some of the prevalent specimens received in frozen section (FS) include breast, head and neck surgery, and neurosurgery specimens [2,3]. The use of intraoperative consultation has increased in recent years, providing important diagnostic information when the patient is on the operating table. The IFS procedure is performed without prolonging operative time and serves as an intraoperative real-time guidance to surgeons to make immediate therapeutic decisions while the patient remains on the operating table. FS during surgery has a great impact on the management of patients’ surgery, which has enormously increased either to avoid irrelevant surgery or to decide the extent of the resection, as evidenced [4].

The accuracy of the FS reports helps in further proper clinical management, and it has emerged as an essential tool. Therefore, evaluation of the performance standard of the FS reports is essential for a tertiary care center for a better therapeutic approach. The FS is most commonly employed in head and neck, skin, and breast cancers, as well as for gastrointestinal and hepatobiliary surgery, intrauterine and ectopic pregnancy, liver transplant surgery, lung lesions, prostate carcinoma, salivary gland tumors, small intestine, and endocrine surgery [5]. Some studies have reported the sensitivity, specificity, and positive and negative predictive values of FS upto 90%, which further justify the application of the IFS technique [6,7]. To assess the specificity, sensitivity, and diagnostic accuracy, FS findings are always correlated with the paraffin sections, while other ancillary tests required include immunohistochemistry, enzyme histochemistry, and immunofluorescence, which are infrequently used [8,9].

Despite their usefulness, like any other test, FS has limitations. Diagnoses are challenging because of inadequate sampling, poor quality of the sections, cautery and freezing process relics, difficulty in identification of certain types of complicated lesions, and the lack of timely availability of required pathological reports or communication between the operating surgeon and pathologist. Furthermore, FS requires dedicated equipment for processing that can often be unavailable at the site with fewer resources.

A cost-benefit analysis was recently done in Iran, which claimed to be the only economic evaluation study regarding IFS in breast cancer patients, and concluded that it is an effective diagnostic method but more expensive. The higher cost of this procedure is due to the time, technical, and personnel resources required to interpret the FS findings at the time of surgeries, together with a controlled performance in making decisions during intraoperative surgeries [10-12].

The efficiency of FS diagnosis can be enhanced by analyzing procedural deficiencies, diagnostic discrepancies, and resolving the underlying causes. Therefore, the present study aims to evaluate the indications for IFS consultation and determine its diagnostic accuracy across major anatomical sites to improve clinical management.

## Materials and methods

Study design and participants

A retrospective study was conducted on all histopathology specimens received for IFS investigation over two years (January 2023 to December 2024) in the Department of Pathology and Cancer Genetics, Kalyan Singh Super Speciality Cancer Institute (KSSSCI), Lucknow, Uttar Pradesh, India. A total of 228 FS samples from patients with various types of malignancies were included in the study. The diagnostic confirmation was done using the formalin-fixed paraffin-embedded (FFPE) hematoxylin and eosin (H&E)-stained tissue sections, prepared from the remnant frozen tissue or from the definitive surgical specimens.

Ethical approval

This study was a retrospective analysis of existing clinico-pathological records without direct patient contact or intervention. Hence, the Institutional Ethics Committee decided that formal ethical approval and informed consent were not required for the present study.

Sample processing and evaluation

The FS was received in a fresh state from the operating theater for examination in a clean and appropriately labelled container accompanied by a requisition form. First, sample identification, type of specimen, and relevant clinical information were noted, including any history of prior treatment.

After confirmation of clinical indication, IFS sampling and grossing were performed immediately to minimize sampling error. Further, samples were sectioned (3-5 µm) using a cryostat (SLEE Medical GmbH, Germany) with temperature settings of -25°C to -35°C and stained with H&E for intraoperative histopathological evaluations. Corresponding tissues were subsequently fixed in 10% neutral buffered formalin, processed into FFPE blocks, and H&E-stained sections were examined for final diagnosis.

Two experienced pathologists independently evaluated all FS cases included in the study as part of routine intraoperative diagnostic practice. Diagnostically challenging or complex cases were reviewed jointly, and a consensus diagnosis was established before final reporting. Therefore, no significant interobserver discrepancy was identified in this study.

IFS diagnoses were compared with the gold-standard FFPE histopathology. Cases were categorized as true positive, true negative, false positive, and false negative. Diagnostic performance parameters were calculated using standard formulas. Concordance between FS and final diagnosis was assessed, and discordant cases were analyzed to determine sampling, interpretational, and technical errors.

In the present study, no cases were deferred during IFS consultation. Accordingly, all cases were categorized as either concordant or discordant in comparison with the final histopathological diagnosis.

Statistical analysis

Categorical variables are summarized as numbers and percentages. Diagnostic performance parameters, including sensitivity, specificity, positive predictive value (PPV), negative predictive value (NPV), overall accuracy, and the kappa coefficient, were calculated with a 95% confidence interval. Statistical analysis was performed by using SPSS software version 16 (SPSS Inc., Chicago, IL, USA).

## Results

**Figure 1:**
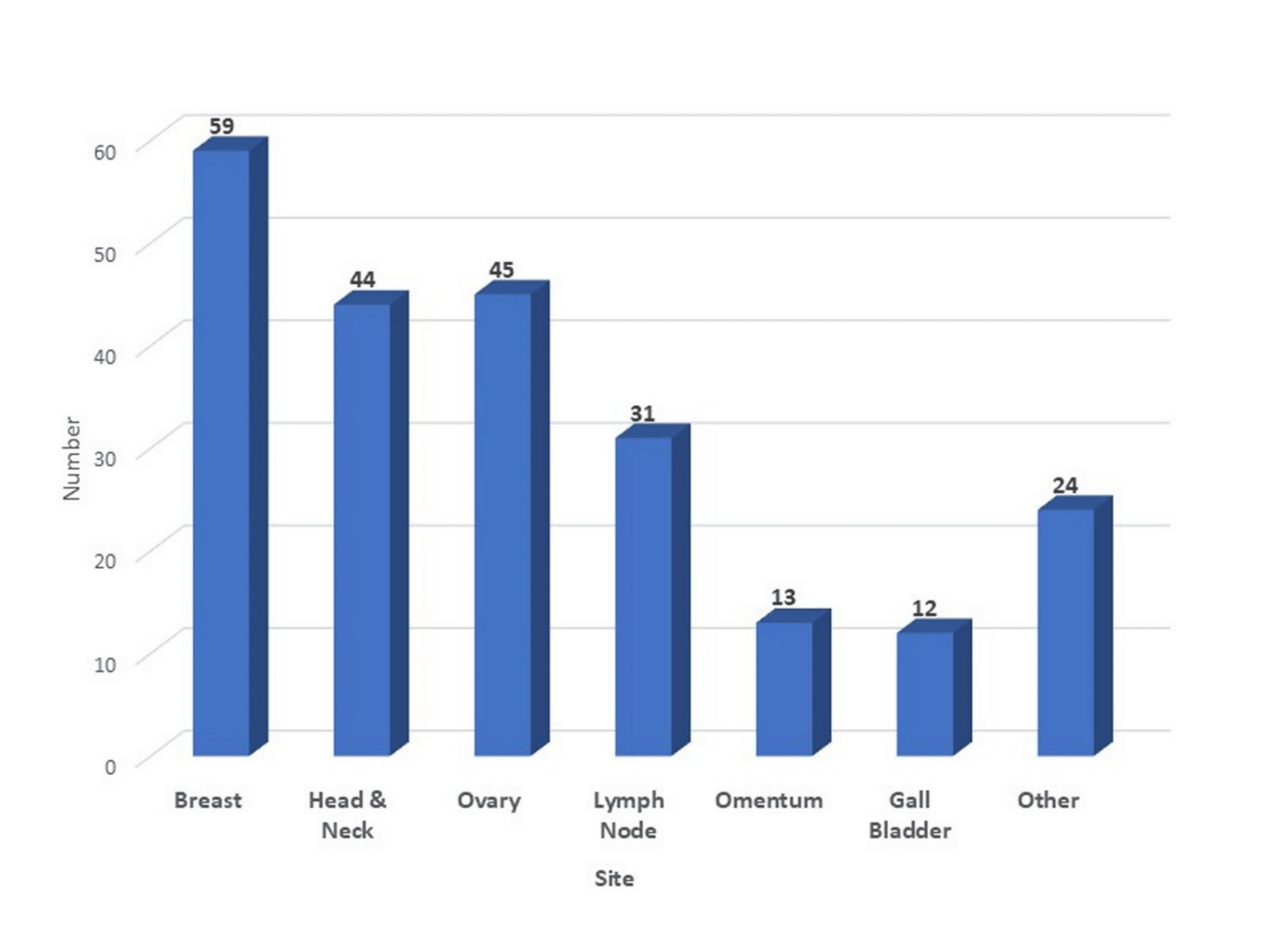
Number of frozen section samples from various anatomical sites.

During the two-year retrospective study, 228 cases underwent FS and FFPE section analysis. Of these, 146 (64%) were females, and 82 (36%) were males, with an age range of 10-74 years. Tissue specimens from various anatomic sites were received for FS examination. The maximum number of FS consultations was requested for the breast (59, 25.8%), followed by the ovary (45, 19.7%), the head and neck (44, 19.3%), lymph nodes (31, 13.6%), omentum (13, 5.7%), and the gallbladder (12, 5.3%). However, 24 (10.5%) other sites included anatomical sites with limited case numbers: six from the uterine cervix, four each from the liver and peritoneum, three from the cystic duct, and one specimen each from the pancreas, ureter cut end, thyroid, eyelid, skin (sole foot), central nervous system, and bone and soft-tissue (Figure 1).

**Figure 2:**
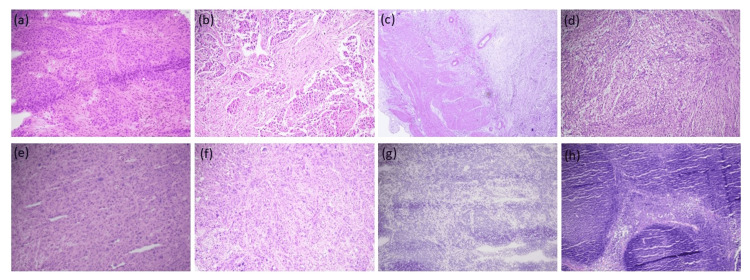
Microphotographs showing comparative diagnoses made by frozen section and histopathology. (a) Frozen section (FS) of breast cancer. (b) Hematoxylin and eosin (H&E) tissue section of breast cancer. (c) FS of xanthogranulomatous cholecystitis. (d) H&E tissue section of xanthogranulomatous cholecystitis. (e) FS of squamous cell carcinoma of the tongue. (f) H&E tissue section of squamous cell carcinoma of the tongue. (g) FS of an uninvolved lymph node. (h) H&E tissue section of an uninvolved lymph node (H&E, ×100).

Concordance and discordance rates between the IFS diagnosis and the final histopathological diagnosis were determined for the above-mentioned six major sites, namely, the breast, ovary, head and neck, lymph nodes, omentum, and gallbladder. Assessment of surgical margins was the most frequent indication for FS examination (107, 46.9%), followed by primary diagnosis (73, 32%) and evaluation of metastasis (48, 21.1%). All cases in our study were analyzed and compared between the diagnosis given on FS and conventional histopathology (Figure 2). The turnaround time for IFS consultation, the mean reporting time was calculated as approximately 22 minutes (range = 15-25 minutes) from specimen receipt to communication of diagnosis.

Table 1 summarizes the diagnostic performance of IFS. Among the 228 cases evaluated, 75 were reported as positive and 153 as negative on FS diagnosis. Further, in the final histopathological examination, 74 cases were true positives, 145 were true negatives, one case was a false positive, and eight cases were false negatives. The diagnostic sensitivity and specificity of IFS were 90.2% and 99.3%, respectively. The PPV was 98.7%, and the NPV was 94.8%. The overall diagnostic accuracy between FS and final histopathology was 96.1%.

**Table 1:** Diagnostic accuracy of frozen section compared with histopathology diagnosis. Sensitivity = TP/(TP + FN); specificity = TN/(TN + FP); PPV = TP/(TP + FP); NPV = TN/(TN + FN); accuracy = (TP + TN)/total cases PPV = positive predictive value; NPV = negative predictive value; TP = true positive; TN = true negative; FP = false positive; FN = false negative; FS = frozen section; CI = confidence interval

		Histopathological diagnosis		Total	Sensitivity	Specificity (95% CI)	PPV	NPV	Accuracy (95% CI)
		Positive	Negative						
Intraoperative FS diagnosis	Positive	74	1	75	90.2%	99.3%	98.7%	94.8%	96.1%
	Negative	8	145	153					
Total		82	146	228					

A total of nine discordant cases were identified, comprising one false-positive and eight false-negative cases. A review of these discordant cases revealed interpretational error in five cases, inadequate sampling in three cases, and technical errors in one case (Table 2).

**Table 2:** Discordant results of frozen section with remnant tissue histopathology and the cause of the error.

Sample type	Frozen diagnosis	Frozen tissue and remnant tissue histology	Cause of discordance	Number of cases
Ovary and omentum	Mature teratoma	Immature teratoma with gliomatosis	Inadequate sampling	1
	Omentum: negative for malignancy			
Gallbladder	Adenocarcinoma	Adenomatous hyperplasia	Interpretation error	1
Ovary	Borderline tumor	Malignant	Inadequate sampling	2
Lymph node	Negative	Positive	Interpretation error due to post-chemo changes	1
Oral cavity: margins	Negative	Moderate dysplasia	Interpretation error	1
Omentum	Negative	Positive	Interpretation error	1
Breast	Negative	In situ component	Technical error	1
Breast: margins	Negative	Positive for lobular carcinoma	Interpretation error	1

## Discussion

FS is a very helpful, highly precise, trustworthy, and rapid diagnostic tool for the optimum management of patients during an intraoperative procedure. This well-established tool not only assists surgeons in confirming the primary diagnosis but also in accurately interpreting and confirming the surgical implications of the patient for improved surgical output, which requires in-depth proficiency. Despite the higher procedural cost, IFS examination is cost-effective by preventing unnecessary surgical interventions, ultimately reducing the overall treatment expenses for patients. Consequently, it contributes to improved surgical decision-making, enhanced clinical efficacy, and therapeutic management [13]. In the current study, we retrospectively reviewed the FS of various anatomical sites to assess the diagnostic accuracy for the optimum clinical management of patients. The average time of reporting of FS in different studies varied from 20 to 25 minutes, and our finding was also in accordance with the reported average time [14,15].

Overall accuracy of FS was 96.1% with a sensitivity of 90.2%, a specificity of 99.3%, a PPV of 98.7%, and an NPV of 94.8%. This is comparable to the results of other published studies, as presented in Table 3 [16-20]. The discordant frequency rate of the present study was 3.9% with nine discordant cases, and this discordance was mainly due to interpretational errors, followed by inadequate sampling and technical errors.

**Table 3:** Comparison of frozen section concordance and discordant rates with various other studies.

Authors	Duration of study (years)	Number of cases	Concordance rate (%)	Discordance rate (%)
Buch et al. [16]	1	122	95.9	4.1
Dayal et al. [17]	1.5	170	95.3	4.7
Patil et al. [18]	2	100	96.9	3.1
Selvakumar et al. [19]	5	132	94.7	5.3
Bharadawaj et al. [20]	2	200	95.5	4.5
Present study	2	228	96.1	3.9

Interpretation errors may result from artefacts of the freezing procedure, causing much damage to the tissue structure of the FS. Inadequate sampling has limitations for the pathologists as they have to interpret the provided sample, and technical errors include poor quality sections and bloated cell morphology [21,22].

On FS examination in the present study, the tumor was diagnosed as a mature teratoma with omentum negative for tumor involvement. However, on permanent histopathological evaluation, extensive additional sections revealed immature neuroectodermal components within the ovarian tumor along with gliomatosis peritonei deposits in the omentum. This discordance was attributed primarily to sampling limitations during intraoperative evaluation.

However, two cases of ovarian tumors were diagnosed as borderline tumors on FS due to the absence of definite stromal invasion in the limited IFS. Malignancy was identified only after additional permanent sections, which demonstrated stromal invasion. These discordant cases clarify that the upgraded diagnosis resulted from additional sampling. The treatment decisions for both patients remained unchanged, as complete surgical resection had already been recommended during the intraoperative consultation.

Moreover, only one case of gallbladder was identified as a false-positive diagnosis on FS. This case was overdiagnosed intraoperatively as an adenocarcinoma; however, permanent histopathological examination established the diagnosis of adenomatous hyperplasia. Adenomatous hyperplasia closely mimics adenocarcinoma on FS due to epithelial hyperplasia with papillary projections and cytologic atypia in the absence of definitive stromal invasion. However, further treatment decisions were based on the final histopathological findings. This case highlights the importance of cautious interpretation during FS evaluation of gallbladder lesions, as false-positive diagnoses may significantly influence intraoperative surgical decision-making and patient management.

In the present study, two cases involving an ileocecal mass and a nasal cavity mass were reported as inflammatory lesions on FS but were subsequently diagnosed as non-Hodgkin lymphoma on permanent sections. The discrepancies were primarily attributable to small biopsy size, freezing artifacts, and suboptimal preservation of cytological and nuclear outlines on FS. Histological features were particularly difficult to evaluate in inflamed, edematous, and fatty tissues, whereas tissue morphology was better preserved in FFPE specimens. The inferior histological quality of FS compared with routine FFPE tissue sections remains a recognized limitation and contributes to false-negative interpretations. Similar observations have been reported in previous studies, emphasizing that section quality, sampling adequacy, and tissue characteristics are major determinants of FS diagnostic accuracy [23,24].

Ultimately, FS interpretation is more challenging than examination of FFPE tissue sections due to technical and morphological limitations. Therefore, appropriate clinical indications and procedural constraints must be carefully considered to ensure accurate intraoperative diagnosis.

FS diagnosis should not replace conventional FFPE histopathology, as it is associated with several potential pitfalls, including limited specimen size, improper orientation of tissue margins, variability in diagnostic accuracy across different anatomical sites, freezing artifacts, inadequate sampling and sectioning, staining quality issues, and dependence on technical proficiency and diagnostic expertise. Consequently, final histopathological diagnosis is always confirmed on corresponding FFPE tissue sections, which allow more extensive sampling of adjacent tissue, and the application of ancillary techniques such as special histochemical stains and immunohistochemistry.

This study has certain limitations. It was a retrospective single-center study, which may limit its generalizability and introduce selection bias. Although the overall sample size was adequate, certain anatomical sites were represented by relatively small numbers, restricting detailed site-specific performance analysis. Additionally, this study did not assess long-term clinical outcomes associated with FS-guided surgical management. Prospective multicentric studies with larger cohorts are warranted to validate the clinical utility of IFS diagnosis further.

## Conclusions

The present study showed that FS is a highly accurate, reliable, and useful diagnostic technique for evaluating a wide range of pathological entities at different anatomic sites. It plays a crucial role in guiding intraoperative decision-making and assists surgeons in selecting the most appropriate therapeutic strategy, thereby improving surgical outcomes. Discordant and deferral rates can be reduced by avoiding technical errors, adequate tissue sampling, interpretational challenges, effective coordination between pathologists and surgeons, and awareness of potential freezing artifacts, particularly in difficult lesions and small biopsies. Optimal utilization of FS ultimately contributes to improved diagnostic precision and better patient care.
